# Identification of Molecular Mechanisms Responsible for the MMP-9-1562C/T Dependent Differential Regulation of Matrix Metalloproteinase-9 Expression in Human Neuron-like Cells

**DOI:** 10.3390/genes14112028

**Published:** 2023-10-31

**Authors:** Sylwia Pabian-Jewuła, Magdalena Ambrożek-Latecka, Aneta Brągiel-Pieczonka, Klaudia Nowicka, Marcin Rylski

**Affiliations:** 1 Department of Translational Immunology and Experimental Intensive Care, Centre of Postgraduate Medical Education, 99/103 Marymoncka Street, 01-813 Warsaw, Poland; magdalena.ambrozek-latecka@cmkp.edu.pl (M.A.-L.); aneta.bragiel@gmail.com (A.B.-P.); 2 Laboratory of Neurobiology, Nencki-EMBL Center of Excellence for Neural Plasticity and Brain Disorders—BRAINCITY, Nencki Institute of Experimental Biology of the Polish Academy of Sciences, 02-093 Warsaw, Poland; nowickaklaudia93@gmail.com; 3 Department of Radiology, Institute of Psychiatry and Neurology, 9 Sobieski Street, 02-957 Warsaw, Poland

**Keywords:** *MMP-9*, MMP-9-1562C/T polymorphism, transcriptional regulation, brain, human neurons

## Abstract

The MMP-9-1562C/T polymorphism exerts an impact on the occurrence and progression of numerous disorders affecting the central nervous system. Using luciferase assays and Q-RT-PCR technique, we have discovered a distinct allele-specific influence of the MMP-9-1562C/T polymorphism on the *MMP-9* (Extracellular Matrix Metalloproteinase-9) promoter activity and the expression of *MMP-9* mRNA in human neurons derived from SH-SY5Y cells. Subsequently, by employing a pull-down assay paired with mass spectrometry analysis, EMSA (Electromobility Shift Assay), and EMSA supershift techniques, as well as DsiRNA-dependent gene silencing, we have elucidated the mechanism responsible for the allele-specific impact of the MMP-9-1562C/T polymorphism on the transcriptional regulation of the *MMP-9* gene. We have discovered that the activity of the *MMP-9* promoter and the expression of *MMP-9* mRNA in human neurons are regulated in a manner that is specific to the MMP-9-1562C/T allele, with a stronger upregulation being attributed to the C allele. Furthermore, we have demonstrated that the allele-specific action of the MMP-9-1562C/T polymorphism on the neuronal *MMP-9* expression is related to *HDAC1* (Histone deacetylase 1) and *ZNF384* (Zinc Finger Protein 384) transcriptional regulators. We show that HDAC1 and ZNF384 bind to the C and the T alleles differently, forming different regulatory complexes in vitro. Moreover, our data demonstrate that *HDAC1* and *ZNF384* downregulate *MMP-9* gene promoter activity and mRNA expression in human neurons acting mostly via the T allele.

## 1. Introduction

MMP-9-1562C/T is a single nucleotide polymorphism (SNP) localized at the -1562 position of the *MMP-9* (Extracellular Matrix Metalloproteinase-9) gene promoter, which substitutes C for T [1]. It has been proposed that this polymorphism may possess a functional impact, as it has been demonstrated that reporter gene expression induced by the T allele is approximately 1.5-fold higher in MALU cells compared to reporter gene expression induced by the allele C [1]. Another hypothesis suggests that the presence of the C allele is responsible for the binding of the nuclear repressor protein to the *MMP-9* promoter, resulting in its reduced transcriptional activity [1,2]. The lack of protein binding to the T allele of the *MMP-9* gene promoter was suggested to enhance its transcriptional activity [1,2]. The exact molecular mechanisms by which the MMP-9-1562C/T polymorphism affects *MMP-9* expression in brain cells might involve different types of transcription factors or other regulatory elements binding to the *MMP-9* gene’s promoter region. Variations in binding affinity due to the C/T change could result in differential gene expression [3]. Numerous pieces of evidence have suggested a correlation between MMP-9-1562C/T polymorphism in the promoter of the *MMP-9* gene and the potential for the onset of many diseases or their progression, which varies from population to population and across different studies conducted within the same population. These connections have been found in neurological diseases like multiple sclerosis (MS) [4,5,6,7], ischemic stroke (IS) [8,9], Guillain-Barré syndrome (GBS) [10], neurodegenerative diseases [11], schizophrenia (SZ) [12], or brain tumors [13].

The data indicated that the *MMP-9* polymorphism, even if it is not the leading player responsible for the development of many diseases, certainly does matter [14]. Until now, no one has demonstrated that *MMP-9* expression is regulated by the MMP-9-1562C/T polymorphism in brain cells. 

We showed for the first time in human neuron-like SH-SY5Y cells that *MMP-9* expression is regulated in an MMP-9-1562C/T-dependent manner. In human neuron-like cells, expression of MMP-9 is higher when the C allele is present. Moreover, *HDAC1* (Histone deacetylase 1) and *ZNF384* (Zinc Finger Protein 384) are regulators of the MMP-9-1562C/T polymorphism.

## 2. Materials and Methods

### 2.1. SH-SY5Y Cell Culture and Differentiation

Human SH-SY5Y neuroblastoma cells (ATCC^®^ CRL-2266) were maintained in 1:1 EMEM/F12 (ATCC, Manassas, VI, USA, Gibco, Paisley, Scotland), supplemented with 10% FBS and 1% penicillin/streptomycin 100X solution (Sigma Aldrich, Burlington, NJ, USA) at 37 °C, 5% atmospheric CO_2_ in a humidified incubator. The ATCC CRL-2266 protocol was implemented for routine neuroblastoma subcultivation procedures. The cells were differentiated according to the protocol described by [15,16] with some modifications. Briefly, 2–3 × 10^4^ cells/cm^2^ were seeded onto culture dishes and allowed to attach overnight in a standard medium with 10% FBS heat-inactivated. The next day, the FBS content of the culture medium was reduced to 5%, and the cells were exposed to 10 µM all-trans retinoic acid (ATRA, VWR, Radnor, PA, USA). The cells were kept under these conditions for five days, with medium changes every second day. On the sixth day of differentiation, the cells were cultured in a standard medium without FBS with the addition of 10 µM ATRA and 50 ng/mL brain-derived neurotrophic factor (BDNF, VWR, Radnor, PA, USA) for an additional 3–4 days. Cells were ready to be used for further experiments.

### 2.2. Immunofluorescence Microscopy

For immunofluorescence microscopy, the SH-SY5Y cultured cells were fixed with a fixing solution (4% paraformaldehyde, 4% sucrose in PBS, filtered) for 6–7 min at room temperature. After fixation, cells were washed three times in warmed PBS with 4% sucrose for 5 min at RT. Then, SH-SY5Y cells were washed three times in PBS, permeabilized for 10 min in PBST (PBS with 0.3% Triton X-100; BioShop Inc, Burlington, ON, Canada), and blocked for 2 h in 3% BSA (Sigma-Aldrich, Schnelldorf, Germany, Burlington, MA, USA) in PBST at RT. The cells were then incubated with mouse monoclonal anti-MAP2 primary antibody (1:1000; M1406, Sigma-Aldrich, Schnelldorf, Germany, Burlington, MA, USA) overnight at 4 °C, washed three times in PBS, and thereafter incubated with the secondary polyclonal antibody conjugated with Alexa Fluor^®^ 488 (goat anti-mouse, 1:500, Invitrogen, Carlsbad, MA, USA) for 2 h in RT. After washing, as indicated previously, coverslips with cells were mounted on glass slides with Fluoromount g mounting medium, dried at room temperature for 5–6 h, and observed with a ZEISS Spinning DISC confocal microscope with a 20× objective lens. Acquisition and analysis were performed with a Java-based image processing program (ImageJ, Version 1.53t).

### 2.3. Preparation of Nuclear Extracts 

Nuclear extracts from SH-SY5Y were prepared according to Bethyl Laboratories’ protocol with slight modifications. Briefly, cells were washed twice with cold PBS and resuspended in buffer A (1 M HEPES, pH 7.9, 1 M KCl, 0.5 M EDTA, 100% Nonindet-40 (Sigma, Burlington, MA, USA), 1 M DTT (Sigma, Burlington, MA, USA), and protease inhibitor cocktail (Sigma, Burlington, MA, USA). The plate with cells was then incubated for 10 min at RT, scraped, and the lysates were transferred to a microcentrifuge tube. Nuclei were pelleted at 4 °C at 13,200 rpm for 3 min. Cytoplasmatic extracts were transferred to the new tube, and nuclei were resuspended in buffer B (1 M HEPES, pH 7.9, 5 M NaCl, 0.5 M EDTA, glycerol, 1 M DTT, and protease inhibitor cocktail). After pipetting, samples were vortexed every 15 min for 2 h, centrifuged at 4 °C at 13,200 rpm for 5 min, and the supernatants were used as nuclear extracts. Protein concentration was estimated by the Pierce Detergent Compatible Bradford Assay Kit (Thermo Scientific, Waltham, MA, USA #23246) using Synergy H4 (BioTek, Winooski, VT, USA). 

### 2.4. Transfection 

**Figure 1:**
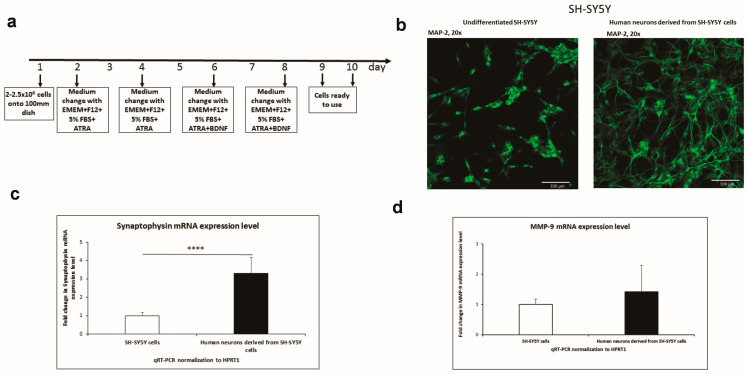
SH-SY5Y human neuroblastoma cell line differentiation into neurons. (**a**) Time table of SH-SY5Y human neuroblastoma cell line differentiation into neurons. (**b**) The photographs show undifferentiated and differentiated SH-SY5Y neuroblastoma cells immunostained with the neuronal marker MAP-2 antibody. Alexa-488 was used to visualize MAP-2. Fluorescence images were obtained using a 20× objective. Scale bar: 100 µm. (**c**) The expression of synaptophysin in SH-SY5Y cells and human neurons derived from SH-SY5Y cells was analyzed by RT-qPCR. The data are presented as a fold change in mRNA expression. The unpaired *t*-test was followed by the D’Agostino and Pearson normality test. Values are given as mean ± SEM (****, *p* < 0.001; *n* = 12). (**d**) The overall expression level of the *MMP-9* (Extracellular Matric Metalloproteinase-9) gene in SH-SY5Y cells and in human neurons. The data are presented as a fold change in mRNA expression. The unpaired t-test was followed by the D’Agostino and Pearson normality test. Values are given as mean ± SEM (*n* = 9).

Undifferentiated and differentiated SH-SY5Y cells were passaged at the same time and then cultured for five days. On the fifth day, cells were trypsinized, counted, and plated in 98-well plates at 4 × 10^4^ cells per well, so they were 70–90% confluent on the day of transfection. Undifferentiated SH-SY5Y cells were plated in a 100 µL standard medium containing serum with antibiotics. In contrast, differentiated SH-SY5Y cells were grown in a medium with 5% FBS, 10 µM ATRA, and antibiotics (5th day of differentiation protocol—Figure 1a). The 6th-day medium was changed to antibiotic-free medium, and 10 µL of the mixture containing 0.3 µL Lipofectamine TM 3000 Reagent (Thermo Fisher Scientific, Waltham, MA, USA), 200 ng appropriate construct (Addgene, Watertown, MA, USA), and 2 µL/µg DNA P3000TM Reagent diluted in 1:1 DMEM/F12 was added to each well. After 6 h after transfection, the medium was changed to standard medium in the case of undifferentiated SH-SY5Y cells and to serum-free medium containing 10 µM ATRA and 50 ng/mL BDNF in the case of differentiated SH-SY5Y cells.

### 2.5. Luciferase Reporter Assay

DNA fragments corresponding to the region containing the MMP-9-1562C/T SNP were amplified by PCR using heterozygous genomic DNA as a template and then cloned into the KpnI and XhoI sites of the pGL3-promoter vector (Promega, Madison, WI, USA). DNA sequence analysis determined the insert’s orientation and allele identity. The undifferentiated and differentiated SH-SY5Y cells were transfected with 200 ng of the appropriate construct using Lipofectamine TM 3000 Reagent. After 24 h, the Dual-Glo^®^ Luciferase Assay (Promega, Madison, WI, USA) was performed according to the manufacturer’s instructions.

### 2.6. Western Blot

The nuclear or cytoplasmatic extracts (10 µg per well) were separated on 12.5% SDS-PAGE in Tris-glycine running buffer (25 mM Tris, 250 mM glycine (pH 8.3), 0.1% SDS) and electroblotted onto polyvinylidene difluoride membrane (Millipore, Burlington, VT, USA) in transfer buffer (48 mM Tris, 39 mM glycine, 0.037% SDS, 20% methanol). Membranes were blocked in 5% nonfat milk/TBST (25 mm Tris-HCl (pH 8.0), 125 mm NaCl, 0.1% Tween 20) for 2 h at RT. Blots were then incubated with an appropriate primary antibody: TBP (TATA) Monoclonal Antibody (51841) 1:1000 (Invitrogen, Carlsbad, United States #MA5-14739); GAPDH 1:5000 (Millipore #MAB374) at 4 °C overnight. Peroxidase labeled anti-mouse IgG secondary antibody (Vector Laboratories, Newark, NJ, USA, # PI-2000) was diluted 1:10,000 in blocking buffer and incubated for over night at 4 °C. Proteins were detected by Western Bright Quantum HRP Substrate (Advansta, San Jose, CA, USA, #K-12042-D10), and chemiluminescence was measured using an Alliance apparatus (UVITEC, Cambridge, UK).

### 2.7. Gel Shift Assay

Gel shift assays were performed using the LightShift^®^ Chemiluminescent EMSA Kit (Thermo Scientific) to visualize DNA-protein complexes. Briefly, 5–10 µg of protein extracts prepared as described above were incubated with 20 fmol of 33-bp nucleotides labeled with biotin and 33-bp non-labeled competitor MMP-9-1562-C or –T (Supplementary Table S1). Supershift assays were carried out by incubating 10 µg nuclear protein extracts with 1 µL of HDAC1 (D5C6U) XP^®^ Rabbit mAb (Cell Signaling Technology, Danvers, MA, USA, #34589) or 0.5 µL of IgG normal Rabbit Ab (Santa Cruze Biotechnology, Dallas, TX, USA, sc-2027 X) (isotype control) for 30 min at RT. Then, DNA probes were added and incubated for 20 min at RT. Reactions with 5 µL ZNF384 Rabbit Ab (Invitrogen, Carlsbad, CA, USA, #PA-52044) or 0.5 µL of IgG normal Rabbit Ab (isotype control) were run at 4 °C. The reactions with competitors (without Ab’s) were incubated for 20 min at RT before the electrophoretic run. DNA-protein and DNA protein-antibody complexes were resolved in 8% polyacrylamide gels in 0.5× TBE buffer (5xTBE: 450 mM Tris, 450 mM Boric Acid, 10 mM EDTA; pH = 8.3) for 1 h with 100 V at RT and transferred to Biodyne™ B Nylon Membrane (Thermo Fisher Scientific, Waltham, MA, USA, #77016) in 0.5× TBE buffer on ice with 100 V for 30 min. The complexes were visualized using Alliance apparatus (UVITEC, Cambridge, UK).

### 2.8. Pull-Down Assay

60 pmol of 33 bp biotinylated nucleotides containing SNP of MMP-9-1562C/T (Supplementary Table S1) were bound to 1 mg of Dynabeads™ MyOne™ Streptavidin C1 (Thermo Fischer Scientific, Waltham, MA, USA). 25 µL of SH-SY5Y nuclear extract was precleared in 1 mL of binding buffer (20 mM HEPES pH 7.6, 1 mM EDTA, 10 mM (NH_4_)_2_SO_4_, 1 mM DTT, 0.2% Tween-20, and 30 mM KCl) in the presence of 1 mg of unbound beads and 288 pmol of 33 bp non-labeled competitor MMP-9-1562-C or –T (Supplementary Table S1). Following magnetic separation, the supernatant was incubated with 1 mg of probe-bound Dynabeads for 30 min at 4 °C. The beads were then washed five times in 1 mL of binding buffer, frozen at −20 °C, and sent for mass spectrometry analysis.

### 2.9. Sample Preparation for Mass Spectrometry Analysis 

Mass spectrometry experiments were performed in the Mass Spectrometry Laboratory at the Institute of Biochemistry and Biophysics, PAS. Proteins were reduced by 1 h incubation with 5 mM tris(2-carboxyethyl) phosphine (TCEP) at 60 °C, followed by 10 min incubation at RT with 8 mM methyl methanethiosulfonate (MMTS). Proteins were digested on beads with 0.5 µgs of trypsin/Lys-C mix (Promega) overnight at 37 °C. Supernatants were transferred to a fresh tube, and beads were washed with the extraction solution (2% acetonitrile, 0.1% formic acid) to ensure maximum peptide recovery. Aliquots were dried on SpeedVac and reconstituted in 40 µL 10 mM HEPES buffer at pH 8.0. The next steps were performed following the SP3 protocol (ultrasensitive proteome analysis using paramagnetic bead technology), with some modifications. Briefly, the SP3 magnetic beads mix was prepared by combining equal parts of Sera-Mag Carboxyl hydrophilic and hydrophobic particles (GE Healthcare, Chicago, IL, USA, 09-981-121 and 09-981-123). The mix was washed three times with MS-grade water and resuspended at a working concentration of 10 µg/µL. Samples were mixed with 16 μL of the prepared bead mix and 1 mL of acetonitrile containing 0.1% formic acid to facilitate peptide binding. Beads were rinsed two times with 1 mL of isopropanol, followed by acetonitrile. Peptides were eluted from the beads by subsequent incubation with MS-grade water and 2% DMSO with sonication during each step. Pulled aliquots were dried in SpeedVac and resuspended in 50 µL 2% acetonitrile and 0.1% formic acid. Peptide concentrations were measured using the Pierce Quantitative Colorimetric Peptide Assay 

### 2.10. Mass Spectrometry and Data Analysis

A total of 1 µg of peptides from each sample was analyzed using nanoAcquity UPLC (Waters) directly coupled to a QExactive mass spectrometer (Thermo Fisher Scientific, Waltham, MA, USA). Peptides were collected on a C18 trap column (180 µm × 20 mm, Waters) with 0.1% FA in water as a mobile phase and transferred to a nanocavity BEH C18 column (75 µm × 250 mm, 1.7 µm, Waters) using an ACN gradient (0–35% ACN in 160 min) in the presence of 0.1% FA at a flow rate of 250 mL/min. Measurements were performed in the data-dependent mode, with the top 12 precursors selected for MS2. Full MS scans covering the range of 300–1650 *m*/*z* were acquired at a resolution of 70,000, with a maximum injection time of 60 ms and an AGC target value of 1 × 10^6^. MS2 scans were acquired at a resolution of 17,500 and an AGC target value of 5 × 10^5^. Dynamic exclusion was set to 30 s. Obtained data were pre-processed with Mascot Distiller software (version 2.6, MatrixScience, London, UK) (Matrixscience, Chicago, IL, USA), and protein identification was performed using Mascot Server 2.5 (Matrixscience, Chicago, IL, USA) against the Homo sapiens protein sequences (20,496 sequences) deposited in the Swiss-Prot database (201,904, 560,118 sequences; 201,292,445 residues). The parameter settings were as follows: enzyme—trypsin, missed cleavages—2, fixed modifications—Methylthio (C), variable modifications–Oxidation (M), instrument—HCD. To reduce mass errors, peptide and fragment mass tolerance settings were established separately for each file after an offline mass recalibration [17]. The confidence assessment was based on the target/decoy database search strategy [18], which provided q-value estimates for each peptide spectrum match. All queries with values >0.01, subset proteins, and proteins identified with one peptide were discarded from further analysis. The mass recalibration, FDR computations, and data filtering were done with Mscan software and developed in-house (http://proteom.ibb.waw.pl/mscan/ accessed on 9 May 2019).

### 2.11. HDAC1 and ZNF384 Silencing with DsiRNAs

Dicer-Substrate Short Interfering RNAs (DsiRNAs) and TriFECTa^®^ Kits (Integrated DNA Technologies, Coralville, IA, USA) were used for RNA silencing. For transfection, SH-SY5Y cells were differentiated as described above. On the fifth day, cells were transferred to 12-well dishes at a density of 450,000 cells/well in the presence of ATRA and antibiotics. The next day, the medium was changed to a fresh one without antibiotics. Cells were transfected using the X-tremeGENE siRNA Transfection Reagent (Roche, Basel, Switzerland) according to the manufacturer’s protocol. 10 nM DsiRNA against HDAC1, ZNF384, positive and negative controls, and 0.5 µg of the pGL3 vector with the promoter of the MMP-9 gene containing the C or T allele were added to the medium. Cells were incubated in the transfection mixture for about 6.5 h, followed by a change of medium with the addition of ATRA, BDNF, and antibiotics. On the seventh day, the medium was changed again to the same medium as above (1:1 EMEM/F12 +ATRA + BDNF + antibiotics). On the eighth day, ca. 48 h after transfection, the cells were flooded with TRIZOL, and the dishes were placed at −80 °C until the subsequent RNA isolation.

### 2.12. RNA Isolation and Reverse Transcription Quantitative PCR

Total RNA was extracted from SH-SY5Y cells using TRIzol^®^ Reagent (Life Technologies, Waltham, MA, USA, #15596018) according to the manufacturer’s protocol. 500 ng of RNA samples were subjected to an RT reaction using the TaqMan Reverse Transcription Kit (Applied Biosystem, Waltham, MA, USA, #N8080234). Real-time PCR analysis was performed with 5× HOT FIREPol EvaGreen qPCR Mix Plus (Solis BioDyne, Tartu, Estonia #08-25-00020) using the LightCycler480 System (Roche Basel, Switzerland). All experiments were performed in triplicate. The CT values were selected within the linear range of amplification, and the comparative CT method was employed to calculate the variances in gene expression. Primers and conditions for RT-qPCR are listed in Supplementary Table S2.

### 2.13. Luciferase Assay after HDAC1 and ZNF384 Silencing

48 h after transfection, luciferase activity was measured using the Dual-Glo^®^ Luciferase Assay System (Promega, Madison, WI, USA) according to the manufacturer’s protocol. Each luciferase construct was assayed in triplicate, and each transfection experiment was repeated at least three times.

## 3. Results

### 3.1. SH-SY5Y Differentiation into Neurons

We used human neuroblastoma SH-SY5Y cells to find out how MMP-9-1562C/T polymorphism regulates *MMP-9* expression. These cells have already been described as having both neuroblast-like and epithelial-like cells [19]. Consequently, we first differentiated SH-SY5Y cells into neurons. For this purpose, we used modified protocols based on the following: [15,20,21] (Figure 1a). It has previously been shown that a combination of serum starvation and the addition of retinoic acid (RA) led to the removal of epithelial-like cells due to apoptosis [21]. In order to verify the differentiation procedure, SH-SY5Y cells were immunofluorescently stained for the neuronal marker MAP-2. According to our optimized procedure, the neuroblastoma cells differentiated into neurons and exhibited significantly more MAP-2 than the undifferentiated SH-SY5Y cells (Figure 1b). Moreover, using RT-qPCR, we revealed that expression of the neuronal marker synaptophysin was significantly higher in differentiated neuronal cells compared to undifferentiated human neuroblastoma cells (Figure 1c). We conducted a further analysis of the overall expression level of the *MMP-9* gene in SH-SY5Y cells and in neurons obtained using the differentiation of SH-SY5Y cells. We have demonstrated that the overall level of *MMP-9* mRNA was slightly higher in mature neurons derived from SH-SY5Y cells as compared to undifferentiated SH-SY5Y cells (Figure 1d).

### 3.2. Allele-Specific Effect of MMP-91562C/T Polymorphism in Luciferase and Gel Shift Assay

**Figure 2:**
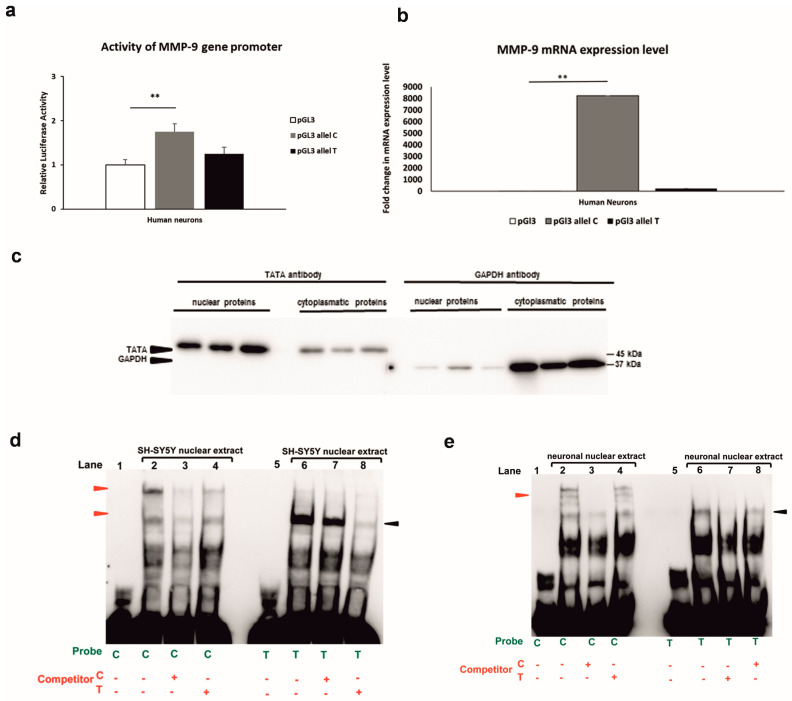
Allele-specific effect of the MMP-9-1562C/T polymorphism in luciferase and gel shift assay. (**a**) Luciferase activities were recorded after transfection of human neurons derived from SH-SY5Y human neuroblastoma cell line with constructs containing either the C or T allele of the *MMP-9* (Extracellular Matrix Metalloptoteinase-9) promoters controlling the luciferase gene. The white bar indicates results obtained after transfection of the *MMP-9* promoter with the empty pGl3 vector. Outliers have been removed, Q = 1%. The Kruskal–Wallis test was followed by the D’Agostino and Pearson normality test. Values are mean ± SEM (**, *p* < 0.05; *n* = 30–35). (**b**) RT-qPCR analysis of *MMP-9* mRNA expression in human neurons transfected with constructs containing the C or T allele of MMP-9-1562C/T polymorphism. The white bar indicates results obtained after transfection of the *MMP-9* promoter with the empty pGl3 vector. Outliers have been removed, Q = 1%. The Kruskal–Wallis test was followed by the D’Agostino and Pearson normality test. Values are mean ± SEM (**, *p* < 0.05; *n* = 10–12). (**c**) Western blot validation of the purity of nuclear and cytoplasmatic extracts isolated from human neurons derived from SH-SY5Y cells. The 10 µg of nuclear and cytoplasmatic fraction protein was loaded on SDS-PAGE and processed using immunoblot analysis with anti-TATA and anti-GAPDH antibodies, which are nuclear and cytoplasmic markers, respectively [22,23] (**d**) Binding of nuclear proteins extracted from the SH-SY5Y cell line to the alleles of MM9-1562C/T was analyzed using gel shift assay. The specifically shifted bands are indicated with the red arrowheads for the C allele and the black arrowheads for the T allele. (**e**) Binding of nuclear proteins extracted from human neurons derived from SH-SY5Y cells to the alleles of MM9-1562C/T analyzed using gel shift assay. The specifically shifted bands are indicated with the red arrowheads for the C allele and the black arrowheads for the T allele.

The luciferase assay was performed to determine whether the −1562C/T polymorphism affects *MMP-9* activity in neuronal cells derived from the SH-SY5Y cell line. We demonstrated that the C allele significantly increased the *MMP-9* gene promoter activity as compared to the control (the empty pGl3 vector) (Figure 2a). The C allele of the MMP-9-1562C/T polymorphism conferred higher activity to the *MMP-9* promoter than the T allele, as we observed in Figure 2a. Next, we assessed the *MMP-9* mRNA expression in human neurons derived from SH-SY5Y cells transfected with constructs containing the C or T allele of the MMP-9-1562C/T polymorphism. RT-qPCR results revealed that the *MMP-9* mRNA expression level was significantly higher in human neurons with the C allele than in control neurons (transfected with the empty pGL3 vector). On the other hand, the level of mRNA expression in human neurons transfected with the T allele was higher compared to the control but lower compared to the neurons with the C allele; the differences were not statistically significant (Figure 2b). These results indicate that the -1562C/T polymorphism affects the *MMP-9* gene promoter activity in human neurons and, importantly, that the activation is allele-dependent. 

In the next step, we used the electrophoretic mobility shift assay (EMSA) to reveal proteins that can regulate *MMP-9* gene expression via differential binding to the alleles of the MMP-9-1562C/T polymorphism [24]. To accomplish this objective, we initially verified the purity of nuclear extracts. The presence of TATA and GAPDH, which are nuclear and cytoplasmic markers, was examined in the isolated protein fractions [22,23]. The Western-blot analysis demonstrated a strong signal for TATA in nuclear extracts and a weak one in the cytoplasmic fractions (left panel, Figure 2c). Whereas GAPDH showed a strong band in cytoplasmic extracts and was almost undetectable in the nuclear protein fraction (right panel, Figure 2c). The high purity of isolated nuclear fractions was confirmed by these results. The EMSA analysis revealed, in the experiment with the C allele, two bands that were completed by the non-labeled C allele but not by the T allele (red arrowheads). In contrast, the gel shift experiment conducted with the T allele revealed a single shifted band, which was completed by the non-labeled T allele but not by the C allele (represented by black arrowheads) in the SH-SY5Y nuclear extract (Figure 2d). The experiment with human neurons derived from SH-SY5Y cells yielded a similar conclusion, with three shifted bands with the C allele, which were completed by the non-labeled C allele, and one band in the T allele, completed by the non-labeled T allele but not by the C allele (Figure 2e). Results from the gel shift assay suggest that different sets of proteins regulate *MMP-9* expression in an MMP-9-1562C/T allele-specific manner (Figure 2d,e).

### 3.3. Identification of the MM9-1562C/T Binding Proteins in Human Neurons

**Figure 3:**
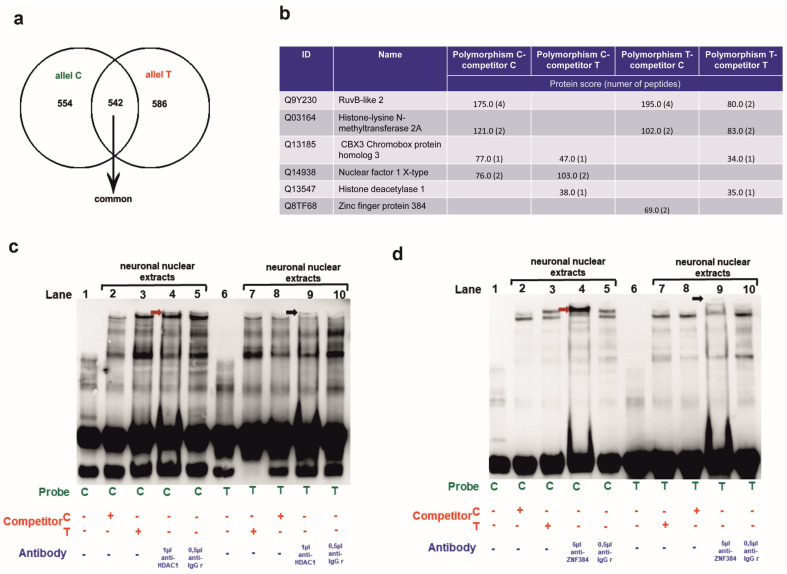
Identification of the MM9-1562C/T binding proteins in human neurons. (**a**) Venn diagram analysis showing the overlap between proteins bound to the C (green) and T (red) allele in the MMP-9-1562C/T polymorphism of the *MMP-9* gene. (**b**) The table depicts the proteins detected using pull-down assay combined with mass spectrometry and selected for EMSA supershift. (**c**) Binding of human neuronal nuclear lysates to the alleles of MM9-1562C/T supershifted with HDAC1 antibody using the EMSA method. A control reaction was conducted with an isotype antibody. The specifically shifted band is indicated with the red arrowhead for the C allele and the black arrowhead for the T allele. (**d**) Binding of human neuronal nuclear lysates to the alleles of MM9-1562C/T incubated with the ZNF384 antibody and subjected to EMSA supershift. A control reaction was conducted with an isotype antibody. The specifically shifted band is indicated with the red arrowhead for the C allele and the black arrowhead for the T allele.

In the subsequent experiment, the pull-down assay followed by mass spectrometry analysis was employed to identify MM9-1562C/T binding proteins in human neurons. We have identified a total of 554 proteins that were bound to the C allele of the MMP-9-1562C/T polymorphism and 586 proteins that were bound to the T allele, of which 542 proteins were shared among the groups (Figure 3a). 

Next, we have selected a few proteins from the data that could be potentially interesting in the aspect of *MMP-9* expression regulation (Figure 3b). It has been reported that *YY1* (Ying Yang 1) is a critical repressor of *MMP-9* transcription in brain neurons [25]. Thus, we checked YY1 protein association networks (String software, version 10.5). The analyses showed Histone deacetylase 1 (HDAC1) and RuvB-like 2 (RUVBL2) as YY1 partners, and we selected these proteins for further studies. Moreover, Matinspector bioinformatics identification of transcription factors capable of binding to the MMP-9-1562C/T polymorphism revealed Zinc finger protein 410 (*ZNF410*) as a transcription factor capable of binding to the C or T allele. Interestingly, using a protein pull-down assay combined with mass spectrometry analyses, we identified a ZNF-410-related DNA binding protein, Zinc finger protein 384 (ZNF384), so we also included this transcription factor in our further studies. The rest of the chosen transcription factors selected for further analyses (Lysine Methyltransferase 2- *KMT2A*, *Nuclear Transcription Factor*, *X-box binding 1-NFX1*, *Chromobox 3-CBX3*) are strongly involved in gene silencing or activation, and therefore they could hypothetically influence *MMP-9* expression. Moreover, the transcription factors *KMT2A* and *CBX3* are involved in chromatin remodeling, similar to *YY1* (Figure 3b). 

We performed EMSA supershift analyses to check whether the selected proteins could bind to the MMP-9 gene promoter. We found that antibodies against four out of six chosen transcription factors (*RUVBL2*, *KMT2A*, *CBX3*, and *NFIX*) did not generate gel shifts. This meant that they did not bind to the promoter fragment of the MMP-9 gene in both the C and T alleles. However, we observed an HDAC1-supershifted band in the C allele-containing samples, indicating HDAC1/the C allele interaction (Figure 3c, lane 4). Regarding the T allele, the band was also visible, but it was localized higher and weaker. This suggests that HDAC1 binds to the T allele less intensely and in the presence of other protein complexes than to the C allele (Figure 3c, lane 9). This may mean that in the case of the T allele, the promoter of the *MMP-9* gene is occupied by other proteins with which HDAC1 forms a complex. Furthermore, it binds to the promoter of the *MMP-9* gene in smaller amounts. In a similar manner, the gel shift assay conducted with the ZNF384 antibody revealed a band shift with the C allele visible in lane 4 (Figure 3d, lane 4) and a weaker and higher supershifted band with the T allele (Figure 3d, lane 9). Therefore, we have found an in vitro interaction of HDAC1 and ZNF384 DNA-binding regulatory proteins with the MMP9-1562C/T polymorphism. We have also shown that it is allele-dependent and strongly visualized with the C allele than the T allele in the experimental setting. Moreover, both HDAC1 and ZNF384 form different in vitro complexes with the C allele than with the T allele. Interestingly, the T allele complexes are larger than those containing the C allele.

### 3.4. Effects Exerted by HDAC1 and ZNF384 on the MMP-9 Promoter Activity and Its mRNA Expression

**Figure 4:**
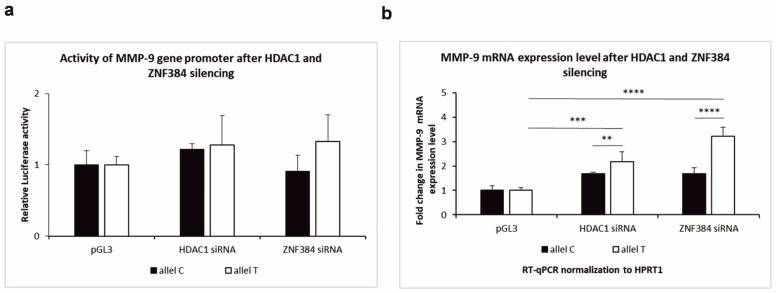
Knockdown of *HDAC1* or *ZNF384* by DsiRNA in human neurons derived from SH-SY5Y cells. (**a**) *MMP-9* activity was measured using luciferase assay after silencing of *HDAC1* or *ZNF384* in human neurons with constructs containing either the C or T allele of *MMP-9* promoters controlling the luciferase gene. A control reaction was conducted with constructs containing the empty pGl3 vector. The Kruskal–Wallis test was followed by the D’Agostino and Pearson normality test. Values are mean ± SEM (*n* = 20). (**b**) *MMP-9* mRNA expression was measured by RT-qPCR after silencing of *HDAC1* or *ZNF384* in human neurons transfected with constructs containing either the C or T allele of the MMP9-1562C/T polymorphism. A control reaction was conducted with constructs containing the empty pGl3 vector. Outliers have been removed; Q = 1%. One-way ANOVA was followed by the Kolmogorov–Smirnov test. Values are mean ± SEM (**, *p* = 0.0030, ***, *p* = 0.0005, ****, *p* < 0.0001; *n* = 6–8).

Next, we asked whether HDAC1 and ZNF384 could influence *MMP-9* promoter activity. Therefore, we knocked down HDAC1 and ZNF384 by DsiRNA in human neurons, and we measured *MMP-9* activity by luciferase assay. As a result, we observed higher activity of the *MMP-9* gene promoter in the presence of the C allele or the T allele as compared to control (human neurons transfected with the empty pGL3 vector) in *HDAC1*-silenced neurons (Figure 4a). Moreover, we found that, in *ZNF384*-silenced neurons, the activity of the C allele of the *MMP-9* gene promoter was lower than in the control (Figure 4a). On the contrary, our data showed that the T allele activity was higher as compared to the control in *ZNF384*-silenced neurons (Figure 4a). Altogether, our results demonstrated that HDAC1 and ZNF384 proteins influence the *MMP-9* promoter activity in a MMP9-1562C/T allele-dependent manner. The data suggest that *HDAC-1* downregulates both the C and the T allele activity, and *ZNF384* upregulates the C allele activity and downregulates the T allele activity in human neurons.

Then, we used RT-qPCR to examine whether *HDAC1* and *ZNF384* influence neuronal *MMP-9* mRNA expression in an MMP-9-1562C/T polymorphism-dependent manner. We knocked down *HDAC1* or *ZNF384* expression by DsiRNA in human neurons derived from SH-SY5Y cells, and we measured the *MMP-9* mRNA level. Interestingly, we observed an elevation of the *MMP-9* mRNA expression for both HDAC1- or ZNF384-silenced neurons with both the C allele or the T allele as compared to control (non-silenced human neurons transfected with the pGL3 vector containing the T or C allele) (Figure 4b, white column). However, the *MMP-9* expression increase was statistically significant only in the T allele but not in the C allele transfected neurons (Figure 4b, black column). As we previously showed using EMSA supershift for the T allele, both HDAC1 and ZNF384 appear to be complex with other yet unidentified proteins, which we did not observe for the C allele. This binding is crucial, and all proteins in this complex appear to regulate the *MMP-9* gene promoter in the presence of the T allele and maintain it at a low level under physiological conditions. However, upon silencing *HDAC1* and *ZNF384*, the expression of the *MMP-9* gene increases dramatically, indicating that *HDAC1* and *ZNF 384* are the main players in this process. It all together suggests that *HDAC1* and *ZNF384* regulate gene expression in an MMP-9-1562C/T-dependent manner and that this regulation is exerted in unstimulated human neurons mostly by their binding to the T allele. Moreover, the data imply that *HDAC1* and *ZNF384* are negative regulators of *MMP-9* gene expression.

## 4. Discussion

This study revealed for the first time the implication of the MMP-9-1562C/T polymorphism on the mRNA expression of *MMP-9* in human neurons. Robert & Pelletier reported that the presence of SNPs in transcriptional regulatory elements influences the mRNA expression of this gene [26]. Therefore, the cytosine (C) > thymine (T) single nucleotide polymorphism at position -1562 in the *MMP-9* promoter may affect the expression level of *MMP-9* mRNA and, consequently, susceptibility to various diseases related to the incorrect expression of this protease. 

This is the first study to examine the role of the MMP-9-1562C/T gene promoter polymorphism in the expression of *MMP-9* in brain cells and related transcriptional regulation. Here, we showed that *MMP-9* promoter activity was almost two-fold higher in the presence of the C allele as compared to the control (human neurons transfected with an empty pGl3 vector). In the case of the T allele, the *MMP-9* promoter activity was 1.4-fold higher as compared to the control, although the differences were not statistically significant. Similarly, the mRNA expression of *MMP-9* varied depending on the MMP-9-1562C/T polymorphism. The *MMP-9* mRNA level was significantly higher when the C allele was present in human neurons as compared to a control. The expression of *MMP-9* mRNA was also observed to be elevated in human neurons transfected with a pGl3 vector containing the T allele in comparison to the control, even though the differences were not statistically significant. Experiments performed by Zhang et al., however, have produced conflicting results. They showed that in MALU cells, the reporter activity of the *MMP-9* promoter was almost 1.5 times greater under the control of the T allele than the reporter activity controlled by the C allele [1]. It is noteworthy that the allele-dependent impact of the MMP-9-1562C/T polymorphism was not demonstrated in the studies conducted on primary amnion epithelial cell cultures, WISH, or THP-1 cells [27]. The discrepancy between their studies and ours in this respect is puzzling. Therefore, we speculate that the mechanism responsible for the differences in the allele-dependent *MMP-9* promoter activity is perhaps cell type-dependent.

Next, we investigated differences in transcriptional regulatory proteins binding to the *MMP-9* gene promoter in relation to the C or T allele. Using the electrophoretic mobility shift assay, we showed two bands in the case of allele C, which were completed by the non-labeled allele C but not by the allele T. In comparison, the experiment with the allele T showed one shifted band, completed by the non-labeled allele T but not by the allele C in purified SH-SY5Y nuclear extracts. A similar conclusion was reached from the experiment with neuronal nuclear extract showing three bands in the case of allele C, which were completed by non-labeled allele C, and one shifted band in allele T. The results suggest that different sets of proteins regulate the transcriptional mechanism in the promoter of the *MMP-9* gene in an allele-specific manner. The results are similar to those obtained by Zhang et al., who showed that the bands present on the C allele were less prominent on the T allele [1]. 

We have extended our experiment further by identifying MMP-9-1562C/T binding proteins in human neurons using the pull-down assay followed by mass spectrometry. The analysis revealed 554 proteins that were bound to the C allele in the MMP-9-1562C/T polymorphism of the *MMP-9* gene and 586 proteins bound to the T allele, of which 542 were shared. After mass recalibration, FDR computations, and data filtering with Mscan software, we have selected proteins for further experiments. Additionally, we performed other bioinformatics analyses that helped us find transcription factors that probably interact with the MMP-9 gene promoter, such as analyses in MatInpector and String software. For the C allele in the *MMP-9* gene polymorphism, the Chromobox protein homolog 3 (CBX3), Nuclear factor 1 X-type (NFIX), and Histone deacetylase 1 (HDAC1) were chosen. These proteins, as shown in Figure 3b, bound to the C allele in the presence of a competitor in the form of an MMP-9 promoter fragment with the T allele. So, proteins that were not specific for the C allele were caught with the T allele. Reports have indicated that *CBX3* is involved in transcriptional silencing in heterochromatin-like complexes where it recognizes and binds histone H3 tails methylated at ‘Lys-9’, leading to epigenetic repression [28]. It is also a repressor in mammalian cells when tethered to DNA [29]. In turn, *NFIX* has DNA-binding transcription factor activity [30,31] and regulates RNA polymerase II transcription [32], while *HDAC1* is responsible for transcriptional repression and gene silencing [33,34]. On the other hand, for the T variant in the -1562C/T polymorphism of the *MMP-9* gene, the following proteins were selected: RuvB-like 2 (RUVBL2), Histone-lysine N-methyltransferase 2A (KMT2A) and Zinc finger protein 384 (ZNF384). It has been reported that *RUVBL2* is a component of the NuA4 histone acetyltransferase complex, which is involved in transcriptional activation of selected genes, mainly by acetylation of nucleosomal histones H4 and H2A [35]. Moreover, it positively regulates transcription by RNA polymerase II [36]. Further, *KMT2A* in the MLL1/MLL complex mediates H3K4me, a specific tag for epigenetic transcriptional activation [22,37,38], and positively regulates transcription [39]. The last one of the selected genes, *ZNF384*, is not well studied; however, it is described as a transcription factor that binds sequence-specific double-stranded DNA [40]. 

We performed EMSA supershift in the presence of RUVBL2-, KMT2A-, CBX3-, NFIX-, HDAC1-, and ZNF384-specific antibodies to investigate whether indeed selected transcription factors might specifically bind to the *MMP-9* promoter. We have discovered that antibodies against RUVBL2, KMT2A, CBX3, and NFIX are unable to bind to the promoter fragment of the *MMP-9* gene in both the C and T alleles polymorphism. However, we observed complexes formed with antibodies directed to HDAC1 and ZNF384 in the C and T alleles when human neurons were the source of nuclear proteins. Interestingly, in the case of the T allele, the supershifts were much weaker as compared to the C allele. This suggests that in the presence of the T allele, HDAC1, and ZNF384 may bind to the promoter of *MMP-9* to a lesser extent and/or in the presence of other transcription factors.

The results described above prompted us to evaluate the influence of *HDAC1* and *ZNF384* depletion on *MMP-9* promoter activity in human neurons. We depleted *HDAC1* and *ZNF384* expression by siRNA for *HDAC1* and *ZNF384* and measured *MMP-9* activity by luciferase assay. The *MMP-9* promoter activity was higher under the control of the T allele after both *HDAC1* and *ZNF384* silencing as compared to control, i.e., cells transfected with an empty pGL3 vector. However, the increase in promoter activity was not statistically significant. As for the C allele, after *HDAC1* knockdown, the *MMP-9* activity was slightly higher compared to control. Opposite, after *ZNF384* silencing, *MMP-9* activity was lower compared to control. Previous data showed that overexpression of *HDAC1* downregulated *MMP-9* promoter activity in HT1080 fibrosarcoma cells in a dose-dependent manner [41]. Surprisingly, after silencing both *HDAC1* and *ZNF384*, we have shown that the *MMP-9* mRNA expression level significantly increased when the T allele was present in the promoter as compared to the control, i.e., cells transfected with the empty pGL3 vector. Although *MMP-9* expression was higher in the case of the C allele compared to the control, this increase was not statistically significant. The above results strongly suggest that *HDAC1* and *ZNF384* play an important role in the regulation of the *MMP-9* gene in the presence of the T allele. Previously performed experiments indicated that knockdown of *HDAC1* suppressed the expression and protein level of *MMP-9* in human breast cancer MDA-MB-321, MCF-7 cells, and the U251, T98G glioblastoma cell line [42,43]. Our data show that after *ZNF384* and *HDAC1* silencing, both promoter activity and *MMP-9* gene expression levels increase significantly, but only in the presence of the T allele. In the case of the C allele, after *HDAC1* and *ZNF384* silencing, there are no major differences in promoter activity or gene expression levels of *MMP-9*. Data obtained by other researchers showed that when *HDAC1* was silenced, promoter activity and *MMP-9* gene expression decreased [41,42,43]. In the case of the T allele, as shown in the EMSA supershift image, HDAC1 and ZNF384 transcription factors bind to the *MMP-9* gene promoter in smaller amounts. Therefore, differences in the activity of the *MMP-9* gene promoter and its expression may depend strictly on a specific allele. Unfortunately, no other studies showing the effect of silencing *ZNF384* on the activity of the *MMP-9* promoter or its expression have been published.

## 5. Conclusions

Together, our present data clearly indicate that promoter activity and expression of *MMP-9* are higher in human neurons under the control of the C allele. Moreover, it can be presumed that two transcription factors, *HDAC1* and *ZNF384*, may play an essential role in regulating *MMP-9* transcriptional activity in human neurons only when the T allele is present in the promoter. Although the mechanism of differential transcriptional regulation of the *MMP-9* polymorphism in brain cells is known, its impact on health, disease progression, and diagnostic or therapeutic methods is still being studied. Furthermore, the expression of *MMP-9* can be influenced by a variety of genetic, epigenetic, and environmental factors, requiring a holistic approach when investigating and implementing therapeutic interventions. As we showed in the case of the T allele, HADC1 and ZNF384 are working with different proteins, and we have identified them. It would be of great interest to further examine them or show epigenetic mechanisms that may be important for the MMP-9-1562C/T-dependent differential regulation of *MMP-9* expression in human neurons. Furthermore, it would be interesting to see if this mechanism is similar to other types of cells.

## Data Availability

The SH-SY5Y cell line was provided by LGC Standards, Ltd., Warsaw, Poland.

## Supplementary Materials

The following supporting information can be downloaded at: https://www.mdpi.com/article/10.3390/genes14112028/s1; Table S1: Name and sequence of oligonucleotide probe used for EMSA, EMSA supershift, and pull-down with mass-spectrometry; Table S2: Primer sequences used for RT-qPCR as well as applied PCR conditions.
