# Supplementary material for: Identification of Molecular Mechanisms Responsible for the MMP-9-1562C/T Dependent Differential Regulation of Matrix Metalloproteinase-9 Expression in Human Neuron-like Cells
Source: Genes (Basel). 2023 Oct 31;14(11):2028. doi: 10.3390/genes14112028 (PMC10671763; doi:10.3390/genes14112028)
Supplement: Supplementary file 1 [file genes-14-02028-s001.zip › genes-2661632-supplementary.pdf]

Supplementary Table S1. Name and sequence of oligonucleotide probe used for EMSA, EMSA *supershift*, and pull-down with mass-spectrometry.

Name of oligonucleotide probe	Sequence
allelCbiotin	5'Biotin-CAG GCG TGG TGG CGC ACG CCT ATA ATA CCA GCT3'
allelCcomplementary	5'AGC TGG TAT TAT AGG CGT GCG CCA CCA CGC CTG3'
allelTbiotin	5'Biotin-CAG GCG TGG TGG CGC ATG CCT ATA ATA CCA GCT3'
polimTcomplementary	5' AGC TGG TAT TAT AGG CAT GCG CCA CCA CGC CTG3'
allelC	5'CAG GCG TGG TGG CGC ACG CCT ATA ATA CCA GCT3'
allelT	5'CAG GCG TGG TGG CGC ATG CCT ATA ATA CCA GCT3'

Supplementary Table S2. Primer sequences used for RT-qPCR as well as applied PCR conditions.

Primers	PCR conditions
F: 5'- CACCATCCGTTGCGGACTTAC-3'	45 cycles of 95°c for 10 sec,<br>60°C for 15 sec,<br>and 72°C for 15 sec
R: 5'- CCTCGGGCAAATGTCTTACCAC-3'	
F: 5'- GTGATGATGAACCAGGTTATGACC-3'	45 cycles of 95°c for 10 sec,<br>64°C for 15 sec,<br>and 72°C for 15 sec
R: 5'- CATCTCCTTCATCACATCTCGAGC-3'	
F: 5'- CATCGCTGTGAATTGGGCTGG-3'	45 cycles of 95°c for 10 sec,<br>60°C for 15 sec,<br>and 72°C for 15 sec
R: 5'- CCT CTG GTG ATA CTT TAG CAG TTC-3'	
F: 5'- 'CGACCCTAACCGAGGAAGGAG-3'	45 cycles of 95°c for 10 sec,<br>60°C for 15 sec,<br>and 72°C for 15 sec
R: 5'- GGTC TTG CCG TCT TTC TGA TGG TC-3'	
